# Modified clomiphene citrate protocol versus gonadotropin-releasing hormone antagonist protocol in *in vitro* fertilization: a propensity score-matched retrospective study on cost and efficacy

**DOI:** 10.3389/fendo.2026.1888569

**Published:** 2026-07-29

**Authors:** Wanshan Zhu, Dongjia Chen, Xuejun Zhan, Qiuhua Li, Shuangyan Deng, Dongyi Chen, Yingling Feng, Junyu Liang, Cuiting He, Xiaoting Shen

**Affiliations:** 1 Department of Reproductive Medicine Center, Guangdong Provincial Reproductive Science Institute (Guangdong Provincial Fertility Hospital), Guangzhou, China; 2 National Health Commission (NHC) Key Laboratory of Male Reproduction and Genetics, Guangzhou, China; 3 Reproductive Medicine Center, The First Affiliated Hospital, Sun Yat-sen University, Guangzhou, China

**Keywords:** clomiphene citrate, cost-effectiveness analysis, GnRH antagonist, *in vitro* fertilization, propensity score matching

## Abstract

**Background:**

The rising prevalence of infertility has substantially increased the demand for *in vitro* fertilization (IVF). While various controlled ovarian stimulation (COS) protocols exist, the gonadotropin-releasing hormone antagonist (GnRH-ant) protocol is valued for its efficacy in preventing premature luteinizing hormone (LH) surges. However, it involves high medication costs and requires frequent injections, which impose a significant burden on patients. Clomiphene citrate (CC), a classic oral ovulation induction agent, offers advantages such as low cost and convenient administration. With the widespread adoption of the “freeze-all” strategy, its potential adverse impact on the endometrium can be mitigated. Recent modifications to the CC protocol, combining it with adequate-dose gonadotropins, aim to balance efficacy and cost. However, well-designed studies directly comparing this modified CC protocol with the conventional GnRH-ant protocol in a general IVF population are lacking, particularly those incorporating comprehensive cost-effectiveness analyses.

**Methods:**

3,157 patients undergoing IVF or intracytoplasmic sperm injection (ICSI) cycles using either the CC protocol or the GnRH-ant protocol were matched 1:1 using propensity score matching (PSM) based on female age, basal follicle-stimulating hormone (FSH), anti-Müllerian hormone (AMH), antral follicle count (AFC), body mass index (BMI), fertilization method, and sperm source. A total of 1,858 patients (929 per group) were included after matching. Embryological parameters and pregnancy outcomes were compared between the two groups, along with cost-effectiveness and sensitivity analyses.

**Results:**

Baseline characteristics were well-balanced after PSM (with almost all standardized mean differences (SMD) < 0.1). The clinical pregnancy rate was comparable between the CC group and the GnRH-ant group (46.26% vs. 41.07%, *P* = 0.113), and the cumulative clinical pregnancy rate was also similar (51.60% vs. 51.79%, *P* = 1.000). The live birth rate (35.48% vs. 31.95%, *P* = 0.268) and cumulative live birth rate (35.48% vs. 38.29%, *P* = 0.398) were also comparable between the two groups. Compared to the GnRH-ant group, the CC group exhibited a higher Day 3 embryo formation rate (66.67% vs. 62.50%, *P* = 0.012), despite having lower numbers of follicles on the day of hCG trigger and retrieved oocytes (9.00 vs. 10.00 and 6.00 vs. 8.00, *P* < 0.0001). No significant differences were observed in the oocyte retrieval rate, oocyte maturation rate, fertilization rate, and blastulation rate between two groups. The cost of COS in the CC group was significantly lower than that in the GnRH-ant group (1,414.70 [741.50-2,915.50] CNY [~198 USD] vs. 7,266.24 [5,747.64-9,011.05] CNY [~1,017 USD], *P* < 0.0001), representing a saving of approximately 5,851.54 CNY (~819 USD; 80.53%). Cost-effectiveness analysis indicated that the CC protocol was superior across all outcome measures, including incremental cost-effectiveness ratios (ICERs) for oocyte, embryo, and clinical pregnancy. One-way sensitivity analysis (OWSA) indicated that the clinical pregnancy rate between the two groups was the most critical factor influencing the ICER for clinical pregnancy. Probabilistic sensitivity analysis (PSA) also suggested that in 98.50% of simulations, the CC protocol was more cost-saving.

**Conclusions:**

The modified CC protocol was associated with ovarian stimulation effects and clinical outcomes comparable to those of the GnRH-ant protocol. Furthermore, it represented a more cost-effective ovarian stimulation strategy, significantly reducing treatment costs while maintaining a clinical pregnancy rate comparable to that of the GnRH-ant protocol.

## Introduction

The rising prevalence of infertility has increased the demand for *in vitro* fertilization (IVF) (1, 2). Advances in assisted reproductive technology (ART) have yielded various controlled ovarian stimulation (COS) protocols, yet no single optimal regimen exists (3–6). The widely used gonadotropin-releasing hormone antagonist (GnRH-ant) protocol offers flexibility and prevents premature luteinizing hormone (LH) surges (7). However, it requires frequent injections and entails high costs, imposing significant economic and psychological burdens (8). In China, despite partial insurance coverage, non-reimbursable COS medication costs remain a major challenge, with expenses ranging from hundreds to tens of thousands of Chinese Yuan (CNY) (9).

Clomiphene citrate (CC) has been a first-line oral ovulation induction agent for over 60 years (10). Its oral convenience, low cost, safety and efficacy are recognized, and it is included in the 2020 European Society of Human Reproduction and Embryology (ESHRE) guidelines (11). As a selective estrogen receptor modulator, CC blocks estrogen receptors, enhancing endogenous gonadotropin (Gn) secretion and synergizing with exogenous Gn during COS (12) and helps suppress the endogenous LH surge (13). Although CC may impair endometrial receptivity, the increasing use of the “freeze-all” strategy mitigates this issue (14). Thus, it represents a promising alternative to the GnRH-ant protocol, preventing premature LH surges while potentially avoiding associated follicular suppression (15).

Recent evidence supports CC’s efficacy in mild stimulation for poor-prognosis patients (16, 17), though its utility is limited by higher cycle cancellation and lower oocyte yield (18). A modified CC protocol with adequate-dose Gn has been proposed to reduce costs and injection burden while maintaining satisfactory oocyte yield and treatment efficacy. Nevertheless, current studies on this modified protocol remain scarce, predominantly focusing on advanced-age women or patients with diminished ovarian reserve (DOR) (19). Well-designed comparisons with the conventional GnRH-ant protocol in general IVF population are lacking. Furthermore, previous studies often lack comprehensive cost-effectiveness analyses or rigorous control of confounding factors.

To address these gaps, we conducted a propensity score-matched (PSM) retrospective study comparing the efficacy and cost-effectiveness of the modified CC and conventional GnRH-ant protocols in an unselected IVF population.

## Materials and methods

### Study design and participants

This retrospective cohort study was conducted at Guangdong Provincial Fertility Hospital with ethics approval (Project Number: (2025) (14); Date: May 20, 2025). Informed consent was waived due to the retrospective nature of the study, and all patient data were anonymized prior to analysis. The study compared two COS protocols: (1) a modified CC protocol with adequate-dose Gn (referred to as the CC protocol) and (2) a conventional GnRH-ant protocol (referred to as the GnRH-ant protocol). Patients undergoing IVF or intracytoplasmic sperm injection (ICSI) cycles using either protocol between January 2022 and August 2025 were eligible for inclusion.

### Propensity score-matching

Propensity scores were estimated using a logistic regression model that included female age, basal follicle-stimulating hormone (bFSH), anti-Müllerian hormone (AMH), antral follicle count (AFC), body mass index (BMI), fertilization method, and sperm source. Patients were matched 1:1 using a caliper of 0.25 standard deviations of the logit-transformed propensity score. All 929 patients in the CC group were successfully matched, with no patient excluded due to inability to find a match. The matching quality was evaluated by calculating the standardized mean differences (SMD) for all baseline covariates, with an SMD below 0.1 indicating adequate balance.

### Ovarian stimulation protocols

**Figure 1:**
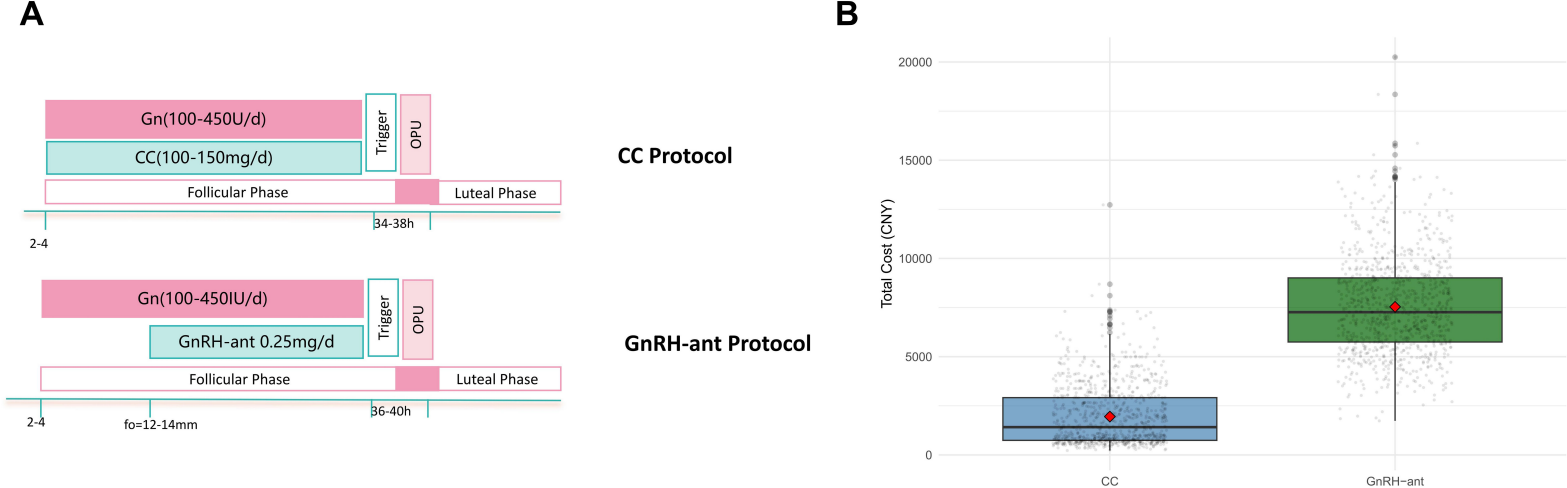
Schematic diagrams of the modified clomiphene citrate protocol and the conventional gonadotropin-releasing hormone antagonist protocol, with a comparison of total drug cost distribution between groups. **(A)** Schematic diagrams comparing the modified CC and GnRH-ant protocols for controlled ovarian stimulation. **(B)** Total drug cost distribution comparison between the groups. Box plots with overlaid individual data points depicting the total drug cost per cycle for the CC group and GnRH-ant group. The red diamond within each box represents the mean value. The median total cost was significantly lower in the CC group compared to the GnRH-ant group (1,414.70 vs. 7,266.24 CNY, P < 0.001). CC, clomiphene citrate; Gn, gonadotropin; GnRH-ant, gonadotropin-releasing hormone antagonist; OPU, oocyte pickup.

Patients in the CC group received CC at a dose of 100–150 mg/day combined with Gn starting on cycle Day 2 or Day 3. The initial Gn dose (100–450 IU/day) was individualized based on factors such as age, BMI, AMH, AFC, and previous ovarian response, and adjusted according to ovarian response, as monitored by transvaginal ultrasound and serum estradiol levels. Patients in the GnRH-ant group received Gn starting from cycle Day 3, with an initial dose of 100–450 IU/day based on the same individualization criteria. GnRH-ant (ganirelix or cetrorelix, 0.25 mg/day) was initiated when the leading follicle reached a diameter of 12–14 mm, and continued until trigger (**Figure 1A**). Final oocyte maturation was triggered with recombinant human chorionic gonadotropin (hCG) (250 μg) or urinary hCG (5,000–10,000 IU) when ≥2 follicles reached ≥18 mm or ≥3 follicles reached ≥17 mm. Transvaginal ultrasound-guided oocyte retrieval was performed 34–38 hours after trigger in the CC group and 36–40 hours after trigger in the GnRH-ant group. Premature ovulation was defined as ovulation confirmed by transvaginal ultrasound prior to scheduled oocyte retrieval.

### Embryo culture and assessment

Oocytes were fertilized via conventional IVF or ICSI, depending on semen parameters and clinical indications. Fertilization was assessed 16–18 hours post-insemination, and normal fertilization was defined by the presence of two pronuclei (2PN). On Day 3, embryos were graded based on cell number, symmetry, and fragmentation (20). Blastocysts were evaluated on days 5–7 according to the Gardner and Schoolcraft grading system (21). Embryo quality scores were calculated using a comprehensive scoring system (**Supplementary Table 1**) that integrated Day 3 and blastocyst morphology parameters, weighted by their respective implantation potential based on published literature. The weighting coefficient for Day 5 blastocysts was set at 1, while the weighting coefficients for Day 3 embryos, Day 6 blastocysts, and Day 7 blastocysts were 0.720, 0.787, and 0.443, respectively (22–24).

### Embryo transfer

Fresh embryo transfer was performed on day 3 or 5 under ultrasound guidance. Frozen-thawed embryo transfer (FET) typically occurred within the first two menstrual cycles after retrieval, with endometrial preparation via a natural or hormone replacement cycle. The number of embryos transferred followed national guidelines, based on patient age and embryo quality (25).

### Outcomes

Primary outcomes comprised ovarian stimulation parameters, oocyte retrieval outcomes, embryological parameters, clinical pregnancy (intrauterine gestational sac with fetal cardiac activity at 6–7 weeks), cumulative clinical pregnancy (from all transfers per retrieval cycle), live birth and cumulative live birth. Secondary outcomes were cost−effectiveness ratios, including cost per oocyte retrieved, per embryo, per clinical pregnancy, and per cumulative clinical pregnancy. Live birth follow-up was successfully ascertained for 99.5% (372/374) of patients in the CC group and 99.7% (726/728) of patients in the GnRH-ant group; the small number of patients lost to follow-up (2 in each group) was unlikely to bias the results.

### Survival analysis

Time to clinical pregnancy (TTCP) was analyzed using Kaplan-Meier curves, with between-group comparisons made by the log-rank test. Cox proportional hazards regression was performed to estimate hazard ratios (HR) with 95% confidence intervals (CI), adjusting for potential confounding factors.

### Cost-effectiveness analysis

Total costs of COS were calculated for each patient based on actual medication usage and local pricing. Costs included all Gn, GnRH-ant (if applicable), and CC (if applicable). All costs were expressed in CNY, with approximate USD equivalents provided in parentheses based on the exchange rate of 1 CNY ≈ 0.14 USD. We calculated cost-effectiveness ratios (C/E) for multiple outcomes: cost per oocyte retrieved, cost per embryo, and cost per clinical pregnancy. The C/E was calculated as total cost divided by the effectiveness measure. Incremental cost-effectiveness ratios (ICER) were calculated as the difference in costs between groups divided by the difference in effectiveness: ICER = (C_1_ - C_2_)/(E_1_ - E_2_), where C_1_ and E_1_ represent the cost and effectiveness of the CC group, and C_2_ and E_2_ represent those of the GnRH-ant group. A negative ICER indicates that the CC protocol is dominant (lower cost and better outcome).

### Markov decision tree model

We employed a Markov decision tree model to compare treatment pathways and outcomes between the two groups. The model traces patient pathways from oocyte retrieval through ET or freeze-all strategy, followed by up to three FET cycles. Numbers represent the percentage of patients following each pathway and clinical pregnancy rates at each transfer cycle.

### One-way sensitivity analysis

One-way sensitivity analysis (OWSA) was performed by varying key parameters ±20% from base-case values. Results were presented in a tornado diagram, with parameters ranked by their impact on the ICER.

### Probabilistic sensitivity analysis

We conducted probabilistic sensitivity analyses (PSA) using Monte Carlo simulation with 1,000 iterations. For each iteration, values for costs and effectiveness measures were randomly sampled from appropriate probability distributions (normal distributions for continuous variables, beta distributions for proportions). Results were plotted on a cost-effectiveness plane, with quadrants representing different scenarios: northeast (higher cost, better outcome), northwest (higher cost, worse outcome), southeast (lower cost, better outcome, indicating dominance), and southwest (lower cost, worse outcome).

### Statistical analysis

All statistical analyses were performed using R software (version ≥4.0.0). Continuous variables were presented as mean ± standard deviation (SD) if normally distributed, or as median with interquartile range (IQR) if non-normally distributed. For continuous variables, between-group comparisons were conducted using independent t-tests for normally distributed data with homogeneous variances (assessed by Levene’s test), or Mann-Whitney U tests for non-normally distributed data or data with heterogeneous variances. For categorical variables, chi-square tests or Fisher’s exact tests (when expected cell frequencies were <5) were used. All statistical tests were two-sided, and *P*-values <0.05 were considered statistically significant.

## Results

### Study population and propensity score-matching

We screened 3,157 patients undergoing IVF/ICSI with either the CC or GnRH-ant protocol. After excluding 100 patients due to missing key baseline data, 3,057 were included in the analysis (CC: n=929; GnRH-ant: n=2,128). The baseline characteristics prior to PSM are presented in **Supplementary Table 2**.

To balance baseline characteristics, 1:1 PSM (caliper=0.25 SD) yielded 1,858 matched patients (929 pairs). The matching quality was excellent, with nearly all variables achieving SMD < 0.1. Key variables showed well-balanced SMDs (female age: -0.087; AMH: 0.062; AFC: 0.073; bFSH: -0.101). The post-matching baseline characteristics are shown in **Table 1**. We also assessed covariate balance before and after matching using Love plots (**Supplementary Figure 1**).

**Table 1:** Baseline characteristics after PSM.

Variable	CC group (N = 929)	GnRH-ant group (N = 929)	*P* value	SMD
Female Age (years)	36.00 [32.00-40.00]	35.00 [32.00-39.00]	0.068	-0.087
Male Age (years)	37.00 [33.00-41.00]	37.00 [33.00-41.00]	0.737	-0.016
bFSH (IU/L)	7.08 [5.82-9.09]	7.03 [5.90-8.60]	0.101	-0.101
bE2 (pg/ml)	158.4 [117.60-211.10]	142.20 [104.90-190.40]	0.008	-0.027
AMH (ng/ml)	1.22 [0.65-2.28]	1.55 [0.89-2.63]	0.077	0.062
AFC	7.00 [4.00-10.00]	8.00 [5.00-11.00]	0.023	0.073
BMI (kg/m²)	22.43 [20.31-24.84]	22.31 [20.50-24.70]	0.698	0.018
Sperm Source				
Husband (n (%))	601 (64.69%)	589 (63.40%)	0.595	-0.013
Donor (n (%))	328 (35.31%)	340 (36.60%)		
Infertility Type				
Primary (n (%))	408 (43.92%)	410 (44.13%)	0.963	-0.002
Secondary (n (%))	521 (56.08%)	519 (55.87%)		
Fertilization Method				
IVF (n (%))	716 (77.07%)	705 (75.89%)	0.584	-0.012
ICSI (n (%))	213 (22.93%)	224 (24.11%)		

PSM, Propensity Score-Matched; CC, Clomiphene Citrate; GnRH-ant, Gonadotropin-Releasing Hormone Antagonist; SMD, Standardized Mean Differences; bFSH, basal follicle-stimulating hormone; bE2, basal estradiol; AMH, anti-Müllerian hormone; AFC, Antral Follicle Count; BMI, body mass index; IVF, *In Vitro* Fertilization; ICSI, Intracytoplasmic Sperm Injection. Mann-Whitney U test for continuous variables. Chi-square test or Fisher’s exact test for categorical variables. SMD, Standardized Mean Difference. Good balance: SMD<0.1.

### Oocyte retrieval outcomes and embryological parameters

The GnRH-ant group had a higher number of follicles on the day of hCG trigger (10.00 vs. 9.00, *P* < 0.001) and a higher number of retrieved oocytes (8.00 vs. 6.00, *P* < 0.001). However, the oocyte retrieval rate was comparable between groups (75% vs. 75%, *P* = 0.777), as was the oocyte maturation rate (91.67% vs. 88.89%, *P* = 0.247) and fertilization rate (82.35% vs. 80.00%, *P* = 0.116). The CC group exhibited a higher Day 3 embryo formation rate (66.67% vs. 62.50%, *P* = 0.012). No significant differences were observed in the blastulation rate (35.70% vs. 33.30%, *P* = 0.081) between two groups. Besides, the GnRH-ant group achieved a higher weighted embryo score (24.00 vs. 27.00 points, *P* = 0.003), calculated based on a comprehensive scoring system incorporating Day 3 and blastocyst morphology parameters (**Table 2**). Of note, the lower weighted embryo score in the CC group was driven by fewer total embryos owing to the fewer retrieved oocytes, not by inferior per embryo quality.

### Pregnancy outcomes

Clinical pregnancy data were available for 1,102 patients (CC: n=374; GnRH-ant: n=728). The clinical pregnancy rate was numerically higher in the CC group (46.26% vs. 41.07%, *P* = 0.113). The cumulative clinical pregnancy rate was nearly identical between groups (51.60% vs. 51.79%, *P* = 1.000). The live birth rate was 35.48% in the CC group versus 31.95% in the GnRH-ant group (*P* = 0.268), and the cumulative live birth rate was 35.48% versus 38.29% (*P* = 0.398), neither showing statistically significant differences between the two groups (**Table 2**). Follow-up for live birth outcomes was complete for 99.5% and 99.7% of patients in the CC and GnRH-ant groups, respectively. Clinical pregnancy rates for cleavage-stage embryo transfers (35.42% vs. 35.41%, *P* = 1.000), blastocyst transfers (57.69% vs. 53.25%, *P* = 0.422), single embryo transfers (50.44% vs. 44.23%, *P* = 0.163), and double embryo transfers (39.73% vs. 37.75%, *P* = 0.754) were all comparable between the CC and GnRH-ant groups. The implantation rate was also similar between groups (36.15% vs. 33.15%, *P* = 0.258) (**Supplementary Figure 3**).

**Table 2:** Embryological parameters and pregnancy outcomes in CC and GnRH-ant groups.

Variable	CC group (N = 929)	GnRH-ant group (N = 929)	*P* value
HCG Follicles	9.00 [5.00-13.00]	10.00 [7.00-14.00]	<0.001
Oocytes	6.00 [3.00-11.00]	8.00 [5.00-12.00]	<0.001
Oocyte Retrieval Rate	75% [54.66%-100.00%]	75% [57.55%-92.86%]	0.777
Maturation Rate	91.67% [75.00%-100.00%]	88.89% [75.00%-100.00%]	0.247
Fertilization Rate	82.35% [66.67%-100.00%]	80.00% [66.67%-100.00%]	0.116
D3 Embryo Formation Rate	66.67% [46.86%-100.00%]	62.50% [42.86%-85.71%]	0.012
Blastulation Rate	35.70% [16.70%-60.00%]	33.30% [16.70%-50.00%]	0.081
Total Weighted Embryo Score (points)	24.00 [10.00-43.00]	27.00 [15.00-45.00]	0.003
Clinical Pregnancy (n/N (%))	173/374 (46.26%)	299/728 (41.07%)	0.113
Cumulative Clinical Pregnancy (n/N (%))	193/374 (51.60%)	377/728 (51.79%)	1.000
Live Birth (n/N (%))	132/372 (35.48%)	232/726 (31.95%)	0.268
Cumulative Live Birth (n/N (%))	132/372 (35.48%)	278/726 (38.29%)	0.398
Severe OHSS (n/N (%))	0/929 (0.00%)	1/929 (0.11%)	1.000

CC, Clomiphene Citrate; GnRH-ant, Gonadotropin-Releasing Hormone Antagonist; OHSS, Ovarian Hyperstimulation Syndrome. Mann-Whitney U test for continuous variables. Chi-square test or Fisher’s exact test for categorical variables. Clinical pregnancy referred to a clinical pregnancy achieved during the first transfer cycle following oocyte retrieval, encompassing both fresh and frozen embryo transfers. Live birth referred to a live birth achieved during the first transfer cycle following oocyte retrieval, encompassing both fresh and frozen embryo transfers.

The incidence of severe ovarian hyperstimulation syndrome (OHSS) was extremely low in both groups. No case occurred in the CC group, while one case was observed in the GnRH-ant group, with no statistically significant difference. Notably, no cycles were cancelled in either group.

TTCP was analyzed using Kaplan-Meier survival analysis among 570 patients who achieved clinical pregnancy (CC: n=193, GnRH-ant: n=377). The median TTCP was 3.0 months in both groups, with no significant difference detected by log-rank test (*P* = 0.77). Kaplan-Meier survival curves showed the cumulative probability of achieving clinical pregnancy over time in the two groups. Shaded areas represent 95% confidence intervals (**Supplementary Figure 2**). Cox proportional hazards regression confirmed no significant difference in the hazard of achieving clinical pregnancy between groups (HR = 0.951, 95% CI: 0.797-1.136, *P* = 0.58).

### Ovarian stimulation parameters and treatment costs

The ovarian stimulation parameters differed significantly between the two groups (**Table 3**). The CC group required a longer duration of gonadotropin stimulation compared to the GnRH-ant group (10.0 vs. 9.0 days, *P* < 0.001). The total gonadotropin dose was also higher in the CC group (2,700.00 vs. 2,400.0 IU, *P* < 0.001). Despite the higher gonadotropin requirements, the total medication cost for COS was significantly lower in the CC group compared to the GnRH-ant group (1,414.70 CNY [~198 USD] vs. 7,266.24 CNY [~1,017 USD], *P* < 0.001), representing a cost reduction of approximately 80.53% (mean difference: 5,851.54 CNY [~819 USD]) (**Figure 1B**). Among the cost, the part of drugs used for LH suppression was reduced by a substantial 96.24% (mean difference: 1,360.74 CNY) in the CC group (62.5 vs. 1,423.2 CNY, *P* < 0.001). Meanwhile, the premature ovulation rate of the two groups was nearly identically at a low level (0.11% vs. 0.32%, *P* = 0.625).

**Table 3:** Cost-effectiveness comparison.

Variable	CC group (N = 929)	GnRH-ant group (N = 929)	*P* value
Gn Duration(days)	10.00 [8.00-11.00]	9.00 [8.00-10.00]	<0.001
Total Gn Dose(IU)	2,700.00 [2175.00-3300.00]	2,400.00 [1,950.00-2,850.00]	<0.001
Cost (CNY)	1,414.70 [741.50-2,915.50]	7,266.24 [5,747.64-9,011.05]	<0.001
LH Suppression Cost (CNY)	62.50 [57.50-67.50]	1,423.24 [1,067.43-1,779.05]	<0.001
Premature Ovulation (n/N (%))	1/929 (0.11%)	3/929 (0.32%)	0.625
C/E for Oocyte (CNY)	235.78	908.28	
ICER for Oocyte (CNY)		2,925.77	
C/E for embryo (CNY)	707.35	2,422.08	
ICER for embryo (CNY)		5,851.54	
C/E for Weighted Score Point (CNY)	58.95	269.12	
ICER for Weighted Score Point (CNY)		1,950.51	
C/E for Clinical Pregnancy (CNY)	3,058.37	17,691.71	
ICER for Clinical Pregnancy (CNY)		-112,849.59	

CC, Clomiphene Citrate; GnRH-ant, Gonadotropin-Releasing Hormone Antagonist; Gn, Gonadotropin. Mann-Whitney U test for continuous variables. C/E represents the cost per unit of outcome. ICER represents the additional cost per additional unit of outcome in the GnRH-ant group compared to the CC group. Negative ICER values indicate that the CC group is dominant (lower cost and better outcome). CNY, Chinese Yuan.

### Cost-effectiveness analysis

The cost-effectiveness analysis demonstrated significant advantages for the CC protocol across all evaluated outcomes (**Table 3**). The cost per oocyte retrieved was substantially lower in the CC group (235.78 vs. 908.28 CNY/oocyte), yielding an incremental cost-effectiveness ratio (ICER) of 2,925.77 CNY per additional oocyte in the GnRH-ant group. For embryo outcomes, the CC group demonstrated superior cost-effectiveness with a cost per embryo of 707.35 CNY compared to 2,422.08 CNY in the GnRH-ant group (ICER = 5,851.54 CNY/embryo). The CC protocol also achieved a lower cost regarding embryo weighted score (58.95 vs. 269.12 CNY/point, ICER = 1,950.51 CNY/point). The cost per clinical pregnancy was markedly lower in the CC group (3,058.37 vs. 17,691.71 CNY/clinical pregnancy). Notably, the CC group demonstrated dominance with lower costs and a higher clinical pregnancy rate, yielding a negative ICER of -112,849.59 CNY per additional clinical pregnancy.

### Decision tree analysis

A probabilistic decision tree was developed to simulate patients undergoing IVF, either using CC protocol or GnRH-ant protocol. The Markov decision tree analysis revealed markedly different treatment strategies between the two protocols (**Supplementary Figure 3**). The CC group adopted a near-universal freeze-all strategy with only 0.1% (1/929) undergoing fresh ET, compared to 23.6% (219/929) in the GnRH-ant group (*P* < 0.001). Among patients with embryos available for transfer, the CC group demonstrated a numerically higher first FET pregnancy rate (46.4% vs 40.9%, *P* = 0.098), although this difference did not reach statistical significance. Second and third FET pregnancy rates were comparable between two groups (34.6% vs 31.9% for second FET, *P* = 0.718; 33.3% vs 37.0% for third FET, *P* = 1.000). Importantly, despite these different treatment pathways, both protocols achieved identical cumulative clinical pregnancy rates. However, the cost differential was substantial: the CC protocol cost 1,414.70 CNY per cycle compared to 7,266.24 CNY for the GnRH-ant protocol (*P* < 0.001), representing an 80.53% cost reduction and establishing CC protocol’s absolute cost-effectiveness dominance.

### Sensitivity analysis

OWSA was performed by varying key parameters by ±20% around their base-case values. The tornado diagram (**Supplementary Figure 4A**) revealed that the number of oocytes in the GnRH-ant group had the greatest impact on the ICER for oocyte (range: 1,625.43 to 14,628.85 CNY/oocyte), followed by the number of oocytes in the CC group (range: 1,828.61 to 7,314.42 CNY/oocyte). The ICER for clinical pregnancy was most affected by the clinical pregnancy rate in the GnRH-ant group (range: -43,669.70 to 193,181.99 CNY/oocyte), followed by the clinical pregnancy rate in the CC group (range: -40,532.69 to 143,911.05 CNY/clinical pregnancy) (**Supplementary Figure 4B**). The cost had relatively smaller impacts on the ICER. Across all scenarios, the CC protocol remained cost-effective.

Monte Carlo simulation with 1,000 iterations was conducted in PSA to assess the robustness of the cost-effectiveness findings. After removing outliers, 977 and 937 valid simulations were analyzed for oocyte retrieval and clinical pregnancy, respectively. The cost-effectiveness plane for oocyte (**Supplementary Figure 4C**) showed that 98.5% of simulations fell in quadrants favoring the CC protocol (39.4% in the southeast quadrant [lower cost, better outcome] and 59.1% in the southwest quadrant [lower cost, slightly worse outcome]). The incremental cost-effectiveness plane for clinical pregnancy (**Supplementary Figure 4D**) demonstrated that 98.5% of the simulations favored the CC protocol, with 52.9% located in the southeast quadrant (indicating lower cost and better outcomes) and 45.6% in the southwest quadrant (indicating lower cost with marginally inferior outcomes). These results confirm the robustness of the cost-effectiveness advantage of the CC protocol.

## Discussion

This study addresses the need for more cost-effective COS strategies by evaluating an alternative to the costly and injection-intensive GnRH-antagonist protocol. Our PSM analysis of 1,858 IVF cycles demonstrates that the modified CC protocol was associated with pregnancy outcomes comparable to those of the GnRH-ant protocol while reducing COS costs by 80.53%. Embryological outcomes were similar between groups, with the CC group exhibiting a higher Day 3 embryo formation rate. Sensitivity analyses confirmed the robustness and distinct cost-effectiveness of the modified CC protocol.

Our finding of significantly lower medication costs in the CC group supports the long-standing view that CC-based protocols are more economical (26–28). While traditional CC minimal stimulation protocols in poor responders or older women show acceptable pregnancy rates and lower cost (29–31), they are limited by fewer oocytes retrieved and higher cancellation rates from premature ovulation or poor response (32, 33), and lower efficacy in non-DOR patients (33–35). The modified CC protocol addresses these shortcomings by administering CC continuously until trigger alongside adequate Gn. This strategy exploits CC’s anti-estrogenic effect to suppress the endogenous LH surge, thereby reducing or eliminating GnRH-ant use (36–39). While a recent analysis in women over 35 found a CC cotreatment protocol to be as effective as a GnRH-agonist long protocol but less costly (40), our study extends this economic advantage to a comparison with the GnRH-ant protocol. Notably, in our cohort, the modified CC group had a lower baseline AFC yet achieved substantially lower drug costs despite requiring higher total Gn and longer stimulation. This is due to the complete replacement of antagonists with oral CC and CC’s potential synergistic effect, which permits the use of more cost-effective Gn such as HMG/uFSH. Patients who opt for the CC protocol are predominantly cost-sensitive, which leads to a preferential use of lower-bioactivity urinary-derived Gn enabled by CC’s synergistic effect, a strategy that further reduces drug costs without compromising overall cost-effectiveness.

A key finding is the comparable incidence of premature ovulation between the CC and GnRH-ant groups. This validates the underutilized yet well-documented mechanism of LH suppression by CC, beyond its recognized role in mild stimulation (13, 41). CC acts primarily at the hypothalamus via its anti-estrogenic effect, increasing FSH release for follicular development while effectively suppressing LH to prevent premature surges and ovulation (42–44). This pharmacodynamic action, consistently shown in historical studies, reliably inhibits LH surges in the late follicular phase (45–48).

Our favorable embryo and clinical outcomes with the modified CC protocol are supported by recent studies. Bhor et al. reported comparable blastulation and pregnancy rates between a low-dose CC (50–100 mg) + 150 IU HMG and a GnRH-ant protocol, despite fewer usable embryos in the CC group (49). Our use of higher CC (100–150 mg/day) and adequate Gn may explain our similar usable embryo rates, suggesting protocol optimization. Lower doses (e.g., 50 mg) risk premature LH rise and suboptimal synergy in DOR patients. Singh et al. reported a trend toward superior outcomes in oocyte donors, with clinical pregnancy rates of 65.94% for CC vs. 57.46% for GnRH-ant (50), aligning with the direction of our results. Most recently, Rs et al. found that in DOR patients, a long CC protocol was effective and patient-friendly, achieving non-inferior oocyte yield and a numerically superior blastulation rate compared to a short 5-day CC + GnRH-ant protocol (51). The apparent inconsistency regarding oocyte yield between their study and ours is likely attributable to population differences. Crucially, both their study and ours consistently observed enhanced blastocyst formation potential, underscoring the CC protocol’s intrinsic benefit to embryo quality.

Although the GnRH-ant group yielded significantly more follicles and retrieved oocytes—potentially attributable to the CC group’s lower baseline AFC—this quantitative advantage did not translate into superior embryo outcomes or pregnancy rates. Conversely, the CC group exhibited a significantly higher Day 3 embryo formation rate. This may be attributed to the more physiological hormonal milieu resulting from CC’s milder LH suppression, which could be more favorable for optimal folliculogenesis and oocyte quality compared to the profound suppression induced by antagonists (52–59). According to the two-cell, two-gonadotropin theory, the synergistic action of FSH and LH is required for optimal steroidogenesis and follicular development (60). CC maintains LH within a physiological range that supports adequate theca-granulosa cell cooperation, whereas profound GnRH-ant-induced LH suppression may compromise oocyte cytoplasmic maturation. While this mechanistic interpretation is biologically plausible, it remains speculative in the context of our observational data and requires prospective mechanistic validation. Excessively low LH levels, due to its short half-life and pulsatile secretion, can impair oocyte quality, while appropriate mid-follicular phase LH levels are beneficial (61–64). The pursuit of this balance was the primary impetus for our study. Given that the natural incidence of a premature LH surge is only about 16.6% (65), we aimed to develop a protocol that provides adequate, not excessive, LH control. Therefore, employing CC to suppress the LH surge enables the maintenance of optimal LH levels without increasing the risk of premature ovulation (66).

Reducing treatment cost and burden is essential for improving access and the chances of parenthood (67). This study demonstrates that the modified CC protocol is a viable, cost-effective alternative to the conventional GnRH-ant protocol for a broad IVF population. It achieves comparable pregnancy rates at markedly lower drug cost, reducing financial pressure. The oral administration of CC also decreases the injection burden and represents a meaningful patient-centered advantage that likely enhancing convenience and compliance. The “freeze-all” strategy in the CC group, necessitated by CC’s potential anti-estrogenic effects on the endometrium, aligns with the modern shift toward segmented IVF cycles and leverages the potential benefits of FET in a non-hyperstimulated endometrial environment (14, 68). The finding that this strategy did not delay time to clinical pregnancy is reassuring and effectively mitigates the historical concern. However, the routine application of this protocol should be considered alongside individual patient characteristics and preferences.

This study has notable strengths, including a large, clinically representative cohort and the use of rigorous propensity score matching to minimize selection bias. A Markov decision tree model was also used to realistically capture all subsequent treatment pathways and their associated probabilities and costs. However, several limitations must be acknowledged. Firstly, the retrospective, single-center design carries an inherent risk of residual confounding from unmeasured factors, including physician preference in protocol selection, patient socioeconomic status and willingness to adopt a freeze-all strategy, as well as temporal changes in laboratory practices and embryology protocols that could not be fully captured in the propensity score model. Secondly, the cost analysis was confined to medication costs, as these remain largely self-paid and represent the most substantial and variable financial burden for patients, whereas procedural costs are subject to heterogeneous insurance coverage that preclude meaningful comparison. Thirdly, although our subgroup analysis demonstrated comparable FET outcomes between groups, endometrial preparation protocols for FET were not distinguished in the current analysis. Fourthly, data on long-term neonatal outcomes were not available. Finally, as the cost calculations are based on the Chinese healthcare context, and drug prices vary considerably across countries due to differences in pharmaceutical pricing policies, procurement systems, and market competition, the absolute savings may not directly translate to other systems with different pricing structures. Additionally, as this was a single-center study conducted in a high-volume Chinese reproductive center, the findings may not be directly generalizable to European or North American healthcare settings. The generalization of our findings requires external validation.

## Conclusion

In conclusion, the CC protocol represents a viable, cost-effective, and patient-friendly alternative to the GnRH-ant protocol in unselected women undergoing IVF in this cohort, and was associated with comparable clinical pregnancy rates at 80.53% lower cost. Its implementation could reduce financial barriers and improve accessibility to ART without compromising treatment outcomes.

## Data Availability

The raw data supporting the conclusions of this article will be made available by the authors, without undue reservation.
