# Supplementary material for: Modified clomiphene citrate protocol versus gonadotropin-releasing hormone antagonist protocol in in vitro fertilization: a propensity score-matched retrospective study on cost and efficacy
Source: Front Endocrinol (Lausanne). 2026 Jul 29;17:1888569. doi: 10.3389/fendo.2026.1888569 (PMC13461314; doi:10.3389/fendo.2026.1888569)
Supplement: Supplementary file 1 [file SupplementaryFile1.docx]

***Supplementary Material***

**Supplementary Figure 1. Standardized Mean Differences Before and After** **Propensity Score Matching**

Comparison of standardized mean differences (SMD) for baseline variables before and after propensity score matching (PSM). The vertical dashed line at SMD=0.1 represents the threshold for good balance. After matching, all of variables achieved SMD <0.1, indicating excellent balance between groups. AFC, antral follicle count; AMH, anti-Müllerian hormone; FSH, follicle-stimulating hormone; sperm_source_H, husband’s sperm; BMI, body mass index; IVF, in vitro fertilization; E2, estradiol.

**Supplementary Figure 2. Kaplan-Meier survival curves for time to clinical pregnancy**

Kaplan-Meier survival curves showing the cumulative probability of achieving clinical pregnancy over time in the CC group (n=193) and GnRH-ant group (n=377). The median time to clinical pregnancy was 3.0 months in both groups. Log-rank test showed no significant difference between groups (P=0.77). Shaded areas represent 95% confidence intervals. CC, clomiphene citrate; GnRH-ant, gonadotropin-releasing hormone antagonist.

**Supplementary Figure 3. Markov decision tree model for cost-effectiveness analysis of ovarian stimulation protocols**

The model simulates patient pathways from oocyte retrieval through either fresh embryo transfer or a freeze-all strategy, followed by up to three frozen-thawed embryo transfer (FET) cycles. Numbers along branches represent the percentage of patients following each pathway and the clinical pregnancy rate at each transfer cycle. The structure highlights markedly different strategic decisions between the CC and GnRH-ant protocols. ET, embryo transfer; FET, frozen-thawed embryo transfer; CC, clomiphene citrate; GnRH-ant, gonadotropin-releasing hormone antagonist.

**Supplementary Figure 4. Sensitivity analyses of the cost-effectiveness evaluation**

(A, B) One-way sensitivity analysis. Key model parameters were varied by ±20%. The incremental cost-effectiveness ratio (ICER) per oocyte was most sensitive to the number of oocytes in the GnRH-ant group. (A). The ICER per clinical pregnancy was most sensitive to the clinical pregnancy rate in the GnRH-ant group (B). The vertical dashed line represents the baseline ICER. (C, D) Probabilistic sensitivity analysis (scatter plots on cost-effectiveness planes). Scatter plot from Monte Carlo simulation (1,000 iterations) showing the distribution of incremental costs (ΔC) and incremental effects (ΔE) for the GnRH-ant group compared to the CC group. The red point represents the base-case result. Quadrants: Northeast (higher cost, better outcome), Northwest (higher cost, worse outcome), Southeast (lower cost, better outcome, indicating dominance), Southwest (lower cost, worse outcome). In both analyses, 98.5% of the simulated iterations favored the CC protocol (points located in the southeast [lower cost, better outcome] and southwest [lower cost, slightly worse outcome] quadrants), confirming the robustness of its cost-effectiveness advantage. ICER, incremental cost-effectiveness ratio; CC, clomiphene citrate; GnRH-ant, gonadotropin-releasing hormone antagonist.

**Supplementary Table 1. Calculation method for the embryo quality score, based on weighted Day 3 and blastocyst morphology parameters.**

| **Day 3** | | | |
| --- | --- | --- | --- |
| The number of cells:  8：4 points  7/9：3 points  6/10：2 points  1-5/11-14：1 point | Symmetry grade  1：3 points  2：2 points  3：1 point | | fragmentation grade:  1：4 points  2：3 points  3：2 points  4：1 point |
| Total points: 11 | | | |
| **Day 5/Day 6/Day 7** | | | |
| Degree of expansion:  4：6 points  5：5 points  3：4 points  6：3 points  2：2 points  1：1 point | | TCM/TE grade:  AA：5 points  AB/BA：4 points  CA/BB/AC：3 points  CB/BC：2 points  CC：1 point | |
| Total points: 11 | | | |

**Supplementary Table 2: Baseline Characteristics Before Propensity Score Matching**

| Variable | CC Group (N=929) | GnRH-ant Group (N=2128) | *P* value | SMD |
| --- | --- | --- | --- | --- |
| Female Age (years) | 36.00 [32.00-40.00] | 33.00 [29.00-37.00] | <0.001 | -0.538 |
| Male Age (years) | 37.00 [33.00-41.00] | 35.00 [31.00-38.00] | <0.001 | 0.317 |
| bFSH (IU/L) | 7.08 [5.82-9.09] | 6.48 [5.49-7.70] | <0.001 | -0.407 |
| bE2 (pg/mL) | 158.4 [117.60-211.10] | 141.65 [105.80-185.72] | 0.523 | 0.015 |
| AMH (ng/mL) | 1.22 [0.65-2.28] | 2.62 [1.39-4.53] | <0.001 | 0.527 |
| AFC | 7.00 [4.00-10.00] | 12.00 [8.00-17.00] | <0.001 | 0.669 |
| BMI (kg/m²) | 22.43 [20.31-24.84] | 21.70 [19.95-24.18] | <0.001 | -0.143 |
| Sperm Source |  |  |  |  |
| Husband (n(%)) | 601 (64.69%) | 1172 (55.08%) | <0.001 | -0.096 |
| Donor (n(%)) | 328 (35.31%) | 956 (44.92%) |  |  |
| Infertility Type |  |  |  |  |
| Primary (n(%)) | 408 (43.92%) | 1146 (53.85%) | <0.001 | -0.099 |
| Secondary (n(%)) | 521 (56.08%) | 982 (46.15%) |  |  |
| Fertilization Method |  |  |  |  |
| IVF (n(%)) | 716 (77.07%) | 1610 (75.66%) | 0.425 | -0.014 |
| ICSI (n(%)) | 213 (22.93%) | 518 (24.34%) |  |  |

CC: Clomiphene Citrate; GnRH-ant: Gonadotropin-Releasing Hormone Antagonist; SMD: Standardized Mean Differences; bFSH: basal follicle-stimulating hormone; bE2: basal estradiol; AMH: anti-Müllerian hormone; AFC: Antral Follicle Count; BMI: body mass index; IVF: In Vitro Fertilization; ICSI: Intracytoplasmic Sperm Injection. Mann-Whitney U test for continuous variables. Chi-square test or Fisher's exact test for categorical variables.

**Supplementary Table 3: Subgroup analysis of clinical pregnancy rates stratified by embryo transfer type**

| Variable | CC Group (N=929) | GnRH-ant Group (N=929) | *P* value |
| --- | --- | --- | --- |
| Implantation (n/N (%)) | 188/520 (36.15%) | 359/1083 (33.15%) | 0.258 |
| Clinical Pregnancy (n/N (%)) | 173/374 (46.26%) | 299/728 (41.07%) | 0.113 |
| Clinical Pregnancy for D3 embryo transfer (n/N (%)) | 68/192 (35.42%) | 176/497 (35.41%) | 1.000 |
| Clinical Pregnancy for blastocyst transfer (n/N (%)) | 105/182 (57.69%) | 123/231 (53.25%) | 0.422 |
| Clinical Pregnancy for single embryo transfer (n/N (%)) | 115/228 (50.44%) | 165/373 (44.23%) | 0.163 |
| Clinical Pregnancy for double embryos transfer (n/N (%)) | 58/146 (39.73%) | 134/355 (37.75%) | 0.754 |

CC: Clomiphene Citrate; GnRH-ant: Gonadotropin-Releasing Hormone Antagonist; Chi-square test or Fisher's exact test for categorical variables. Clinical pregnancy refered to a clinical pregnancy achieved during the first transfer cycle following oocyte retrieval, encompassing both fresh and frozen embryo transfers. Implantation was defined as the number of gestational sacs observed on ultrasound.
